# Vascular Access Devices for Stem Cell Transplantation: A Review of Catheter Types—A Crucial Step Towards the Enhancement of Patient Care

**DOI:** 10.3390/cancers17203325

**Published:** 2025-10-15

**Authors:** Sławomir Milczarek, Piotr Kulig, Oliwia Piotrowska, Alina Zuchmańska, Martyna Brzosko, Bogusław Machaliński

**Affiliations:** 1 Department of Hematology and Transplantology, Pomeranian Medical University, 70-252 Szczecin, Poland; 2 Department of General Pathology, Pomeranian Medical University, 70-111 Szczecin, Poland; 3 Pharmaceutical Facility of Pomeranian Medical University, Pomeranian Medical University, 70-252 Szczecin, Poland

**Keywords:** vascular access, stem cell transplantation, PICC, CICC

## Abstract

Central venous access devices (CVADs) play a pivotal role in managing stem cell recipients, providing reliable access for the administration of chemotherapy, blood products, progenitor infusion, parenteral nutrition, and other crucial treatments. This review critically evaluates the various types of CVADs commonly employed in transplant settings, examining their indications, complications, and best practices to enhance patient outcomes. Moreover, it emphasizes the significance of broadening the selection algorithm for vascular devices and incorporating patient expectations and comfort into routine clinical practice.

## Simple Summary

Stem cell transplantation necessitates versatile and diverse vascular access to facilitate intensive treatment. For autologous and allogeneic transplantation with reduced intensity conditioning (RIC) or non-myeloablative (NMA) conditioning, peripheral access is preferable. For allogeneic transplantation with myeloablative conditioning (MAC), consideration should be given to using a central venous catheter (CICC) or two peripherally inserted central catheters (PICC). CICC is the preferred device when the peripheral vasculature is not optimal for a PICC and when a complicated procedure is anticipated.

## 1. Introduction

Hematopoietic stem cell transplantation (HSCT) is the standard of care for various malignancies and nonmalignant conditions. The procedure is complex, and the management of patients scheduled for HSCT requires the insertion of a multipurpose venous catheter. This catheter is essential for administering chemotherapy, antibiotics, and nutrition, as well as for the transfusion of stem cells and blood products and sample collection. Therefore, central venous access devices (CVADs) are critical in hematological care and directly impact patient outcomes. Understanding various issues such as insertion techniques, management, and potential complications is vital in enhancing overall outcomes and improving the quality of life for stem cell recipients.

The American Society of Clinical Oncology (ASCO) established evidence-based clinical practice guidelines for the care of central venous access devices in 2013 [1]. Subsequently, similar recommendations were published by the European Society for Medical Oncology (ESMO) in 2015 [2]. These guidelines offer crucial recommendations on antibacterial prophylaxis and CVAD maintenance, highlighting areas with insufficient evidence to inform practice, including the lack of evidence to recommend a specific device type or insertion site and discouraging the use of the femoral site. Both guidelines emphasize the importance of selecting the appropriate device based on treatment duration and patient-specific factors. They also advocate for the use of single- or double-lumen CVADs whenever feasible while cautioning against the use of a subcutaneous port for patients requiring intensive therapy, such as hematopoietic cell transplantation recipients, as it may not meet therapy demands. However, the applicability of these recommendations to intensively treated hematological patients remains uncertain due to the lack of prospective data.

The ASCO recommendations stress the importance of well-trained providers in inserting CVADs and advocating for standardized clinical care bundles. Several randomized controlled trials (RCTs) have validated the effectiveness of CVAD bundles in decreasing the incidence of central line-associated bloodstream infections (CLABSI). Proper CVAD maintenance is also crucial to prevent malfunctioning and life-threatening complications, such as catheter-related bloodstream infections due to occlusion, dislodgement, kinking, rupture, and thrombosis. Therefore, adhering to maintenance protocols ensures CVADs’ proper functioning and safety.

In October 2024, the World Congress on Vascular Access (WoCoVA) Foundation, in collaboration with the Global Vascular Access Network (GloVANet), proposed a revised terminology for central vascular access devices (CVADs) [3]. Subsequently, in December 2024, an international expert consensus was published, focusing on the optimal selection and management of central vascular access devices for patients diagnosed with cancer [4]. This expert panel provided comprehensive recommendations addressing various aspects, including the indications for using CVADs, the available CVAD options, factors influencing the decision to select a port over an external catheter, and the recommended flushing frequency when the CVAD is not in active use. Summary of frequently used intravascular devices is presented in Table 1.

While the panel emphasized the importance of patient preference in determining optimal vascular access, no groundbreaking recommendations were introduced. Notably, the consensus applies broadly to the diverse population of oncology patients. However, it is essential to highlight that specific guidelines addressing patients undergoing hematopoietic stem cell transplantation were not included in the recommendations, indicating an area requiring further research and consideration in future guideline development.

Despite extensive retrospective studies and meta-analyses of data on complications among different devices, there have been no updates on CVAD recommendations tailored explicitly to intensively treated hematology patients. The available published data do not offer specific recommendations that can be universally applied to all hematology patients due to the variability in the severity of patient illness, different insertion and site care protocols, and the testing of multiple devices.

In this review, we specifically focus on stem cell recipients. This vulnerable population often experiences a complex interaction between hematology and intensive care. Therefore, individuals undergoing HSCT require a tailored approach to ensure favorable clinical outcomes. Given the lack of specific CVAD recommendations for stem cell recipients, we aim to provide an overview of available vascular access options and offer informed guidance on device preferences based on existing literature and our single-center experience.

## 2. Review Methodology

This narrative review was based on a structured literature search in PubMed, Embase, and Scopus databases, covering publications from January 2010 to June 2025. Search terms included combinations of “vascular access,” “central venous catheter,” “PICC,” “CICC,” “TIVAD,” and “stem cell transplantation.” We included peer-reviewed studies involving adult HSCT recipients, focusing on randomized trials, cohort studies, and registry data. Pediatric-only studies, case reports, and non-English publications were excluded.

Data extraction focused on device type, insertion site, complications (CLABSI, CRT), dwell time, removal rates, and patient comfort. Although no formal risk-of-bias tool was applied, transplant-specific evidence was prioritized.

For transparency, we categorized evidence as HSCT-specific (derived from HSCT cohorts) versus indirect (from oncology—general or mixed populations) and provided absolute rates per 1000 catheter-days where available [8,9,10,11].

## 3. Classification and Types of Vascular Access Devices

CVAD can be designated by its pathway to the vessel, site of insertion, composition, or special features (e.g., silver coating, Dacron cuff, or antibiotics impregnation).

Vascular Access Devices Overview:

• Duration of access:
∘ Short-term (few days or few weeks);
∘ Medium-term (up to 3–4 months);
∘ Long term (>3–4 months).
• Site of insertion:
∘ PICCs (Peripherally inserted central catheters) are inserted into deep veins of the arm (basilic vein, brachial veins, brachial tract of the axillary vein) or the cephalic vein at the arm.
∘ CICCs (Centrally inserted central catheters)—inserted into deep veins of the supra-clavicular area (internal jugular vein, brachiocephalic vein, subclavian vein, deep tract of the external jugular vein) or of the infra-clavicular area (thoracic tract of the cephalic vein, thoracic tract of the axillary vein).
∘ FICCs (Femorally inserted central catheters)—inserted into veins of the lower limb (common femoral vein, superficial femoral vein, saphenous vein).
∘ TIVAD Totally implantable venous access device (or port).
▪ Chest ports, also known as CICC ports.
▪ Femoral ports, also known as FICC port.
▪ Brachial ports, also known as PICC ports, also known as arm ports.
• Number of lumens (single, double, or multi-lumen).
• Characteristic of tip (open tip or valve tip).
• Materials to reduce complications (e.g., silicone or polyurethane catheters impregnated with heparin, antibiotics, or silver).

Types of Vascular Access Devices:

1. PVAD—peripheral venous access devices:
  - SPC—Short peripheral catheter;
  - LPC—Long peripheral catheter (also known as mini-midline or short midline);
  - MC—Midline catheter (also known as midclavicular catheter).

2. CVAD—central venous access devices:
  - PICC—Peripherally inserted central catheters;
  - CICC—Centrally inserted central catheter;
  - FICC—Femorally inserted central catheter;
  - TIVAD—Totally implantable venous access device (or port).
    i. Chest ports, also known as CICC ports;
    ii. Femoral ports, also known as FICC ports;
    iii. Brachial ports, also known as PICC ports, also known as arm ports.

3. Tunneled CVAD:
  - Tc—Tunneled, and cuffed: Tc-CICC, Tc-PICC, Tc-FICC;
  - Tnc—Tunneled, but non-cuffed: Tnc-CICC, Tnc-PICC, Tnc-FICC.

## 4. Catheter Lifespan

### 4.1. Short-Term and Medium-Term Devices

Short-term non-tunneled catheters are commonly used in stem cell transplantation or intensive chemotherapy. Recognizing that the practices surrounding their use may exhibit variability across different medical centers is essential. These catheters provide several advantages, including straightforward placement, rapid access, and the elimination of complex surgical procedures typically required for implantation. CICCs and PICCs can be classified as short-term CVADs, with PICCs providing the potential for a prolonged lifespan if necessary.

Multi-lumen CVAD catheters are essential for navigating complex medical procedures with anticipated adverse outcomes. They offer multiple lumens for therapeutic interventions, sample collection, and measurement of central venous pressure (CVP). Upon CLABSI suspicion, the treating physician can easily remove the catheter. Studies have highlighted that adherence to strict aseptic techniques and proper maintenance can minimize the infection risk associated with non-tunneled CVADs. Additionally, studies comparing different types of central venous access devices found that while non-tunneled CICCs have a higher risk of infection than other CVADs, the overall infection rates are manageable with proper care, making them viable for short-term use [1,12,13,14].

### 4.2. Long-Term Devices

Long-term central venous access devices (CVADs) are seldom utilized exclusively for stem cell transplantation. Both tunneled CVADs and TIVADs fall under this category of vascular devices. Typically, these devices are surgically implanted as a proactive measure before commencing chemotherapy, particularly if vascular complications are anticipated or if the patient is scheduled for prolonged treatment. It is noteworthy that long-term devices offer a reduced risk of central line-associated bloodstream infection (CLABSI) compared to CICCs. They are seldom implanted immediately preceding the procedure, as this necessitates additional hospital stays, and most complications, including bleeding, occur during the early post-procedural period [15]. A prospective study by Kakkos et al. [16] found that short intervals between port implantation and high-risk chemotherapy regimen initiation significantly increased the complication-related removal rate. This may be explained by insufficient tissue healing and port stabilization when chemotherapy is initiated too soon after implantation, increasing the risk of early complications and device removal. Due to reduced lumen in TIVAD, providing intensive supportive treatment or managing immunosuppression levels may present challenges. Moreover, introducing total parenteral nutrition could elevate the risk of catheter dysfunction due to the accumulation of deposits in the tubes of the nutritional mixture [5]. Additionally, if CLABSI is diagnosed, removal is more complicated than CICC.

On the other hand, comparisons of different central venous access devices have shown that while tunneled catheters and ports are preferred for long-term use, non-tunneled CVADs are suitable for short-term needs, balancing efficacy and patient comfort effectively [8,17,18,19]. Non-tunneled CVADs are typically removed after the completion of therapy at the bedside. In contrast, the removal of TIVADs and tunneled CVADs is usually performed in the distant term of transplantation, after an increase in platelet count, and involves additional hospitalization and surgical intervention. While difficult removal is uncommon, the operators must be aware of the issue while removing long-indwelling TIVADs and those with subcutaneous leakage [20]. These distinctions in removal timelines underscore the importance of considering the timing and specific patient-related factors when managing vascular access devices in the context of stem cell transplantation.

## 5. Material Considerations

Catheter materials must ensure biocompatibility, chemical stability, kink resistance, and tensile strength [21,22]. Most CVADs are made of polyurethane (PU) or silicone (SI); legacy materials (PVC, PE, PTFE) are rarely used [23]. PU provides higher pressure tolerance and is standard for power-injectable PICCs, while SI offers superior biocompatibility but may be more prone to mechanical fatigue [21,22].

Infection/thrombosis trade-offs:

− In ports (TIVADs), a large series report higher infection and thrombosis with PU vs. SI, though SI shows more mechanical failures [24].
− In PICCs, older reviews suggest similar or slightly lower infection risk with PU, but outcomes depend more on lumen size, catheter-to-vein ratio, and maintenance bundles than on material alone [25].

Adjunct technologies: Antimicrobial or silver coatings and chlorhexidine-impregnated dressings significantly reduce CLABSI by inhibiting biofilm formation [25,26,27].

HSCT context: No randomized HSCT-specific trials compare PU vs. SI; therefore, recommendations should focus on device type, insertion technique, lumen minimization, and strict bundle adherence, rather than material choice [1].

Detailed comparative data by device/material, lumen number, and insertion site—including absolute CLABSI/CRT rates and IRR/CI—are summarized in Table 2.

## 6. Types of CVADs

Typically, stem cell recipients require multipurpose venous catheters, allowing simultaneous infusion of physically incompatible drugs and blood sample collection. For this purpose four types of VAD are used, depending primarily on transplant center practice and vascular team availability:

### 6.1. Centrally Inserted Central Catheters (CICCs)

CICCs are a central venous access device that provides short- to medium-term venous access. Typically, a CICC is percutaneously inserted directly into a large central vein, such as the subclavian vein or internal jugular vein, using a conventional Seldinger technique, often with ultrasound guidance. The catheter is usually made of flexible materials like polyurethane, multiple lumens (channels), and optionally an antibacterial coating. These catheters are relatively short compared to tunneled or implanted devices, as they do not require a tunnel through the subcutaneous tissue.

Non-tunneled CICCs are generally used for short- to medium-term applications, typically ranging from a few days to a few weeks. They are commonly used when immediate central venous access is needed, such as for administering chemotherapy, intravenous antibiotics, fluids, or total parenteral nutrition (TPN).

CICCs remain the preferred vascular access device in most transplant centers due to their widespread availability and operator preference. While excellent for multimodal treatment, they are unfortunately limited by severe insertion-related mechanical complications, a high catheter-related infection rate, and patient discomfort [24,38].

### 6.2. Peripherally Inserted Central Catheters (PICCs)

PICCs are central venous access devices that provide short- to long-term venous access.

PICCs are inserted percutaneously using the modified Seldinger technique into the peripheral vein of the non-dominant upper arm under ultrasound guidance. When placed successfully, the catheter should end in the cavo-atrial junction. The basilic vein is the recommended insertion location, and the arm’s medial distal part is the preferable puncture site. The catheter/vein ratio should not exceed 45%, as stated by the Infusion Therapy Standards of Practice [6].

PICCs are flexible, thin, and made from silicone or polyurethane. They often have multiple lumens. Their length is adjusted to the puncture site before insertion, making them more tailored to patients. They are suitable for extended periods, from several weeks to several months, and can be used for long-term treatments, such as chemotherapy or long-term antibiotics.

It is ideal for patients needing frequent or prolonged venous access, such as those undergoing stem cell transplantation or intensive chemotherapy, receiving total parenteral nutrition (TPN), or requiring long-term antibiotic therapy. Due to its smaller size (typically 4–5 French) and lumen exit sites, treatment of critically ill patients and immune suppression measurement may be more challenging than CICC. Studies have reported elevated levels of calcineurin inhibitors in blood drawn from PICCs, potentially attributed to drug absorption on the catheter walls [39].

The outstanding benefits of peripherally inserted central catheters (PICCs) encompass the minimal occurrence of insertion-related complications, reduced rate of catheter-related infections, adaptability, patient comfort, and customizable lifespan. Conversely, PICCs are associated with drawbacks such as catheter-related thrombosis and insertion intricacy, necessitating the expertise of operators and rigorous maintenance. Furthermore, to mitigate potential complications, PICCs must be tailored to individual patients’ vasculature and anticipated clinical treatment trajectory.

### 6.3. Tunneled CVADs

Tunneled CVADs travel under the skin, away from the venous entry point, before leaving the skin. A cuff secures the catheter within the subcutaneous tissues. The preferred vessel for insertion is the right internal jugular vein (IJ), followed by the left IJ. The devices are made of polyurethane elastomers or silicone and may remain in the vein for up to several months [40]. Therefore, they are often implanted in hematology patients for long-term chemotherapy administration.

Tunneled Central Venous Access Devices have most of the advantages of short-term catheters, including lower infection rate [41] and Catheter-Related Thrombosis (CRT) [42], but are difficult to implant, requiring surgical implantation and removal—which presents a predicament in cases of CLABSI.

Tunneled CVADs allow for the continuation of intravenous treatment following discharge from the hospital. This type of catheter can also be utilized for hemodialysis or other cytapheresis procedures if needed [43].

### 6.4. TIVAD—Totally Implantable Venous Access Devices

Totally Implantable Venous Access Devices (TIVAD) are a common type of vascular catheter in outpatient oncology settings. These devices are typically subcutaneously implanted on the chest wall, although alternative anatomical sites, such as the thigh or arm, also serve as viable implantation locations. TIVAD configurations may consist of one or two lumens, accessed via specialized atraumatic needles designed to preserve the integrity of the silicone septum, thus minimizing the risk of complications associated with catheter usage. Additionally, their use extends the life of the port and prevents thrombosis and catheter-related infections [43].

Constructed from materials such as polyurethane or silicone, TIVAD can remain implanted for extended periods, making them suitable for patients requiring chronic intravenous therapies, including chemotherapy and blood transfusions. Their subcutaneous nature allows patients to engage in daily activities, including aquatic pursuits, without the concern of catheter-related infections. Notably, the infection rate associated with vascular ports is low, reported at 0.1 bloodstream infections per 1000 catheter days, underlining the safety and efficacy of TIVAD in clinical practice [9].

## 7. General Principles and Indications

When selecting an appropriate CVAD, factors to consider are the intended purpose (e.g., type of transplant), the expected catheter lifespan, the anticipated risk and clinical course of the procedure, maintenance of the device, and patient preference. It is widely acknowledged that delivering chemotherapy medication via adequate central venous access reduces the risk of infusion-related complications [1,2].

Moreover, the ideal vascular device should enable continuous infusion, administration of physically incompatible drugs in high doses, infusion of stem cells, frequent blood sampling, transfusion support, and total parenteral nutrition (TPN) [44]. However, the misconception that “the bigger the lumen, the better the performance” and “more lumens, easier management” has led to the demand for large-bore and/or multiple-lumen catheters for all patients. Research has shown that large-bore vascular access devices (VADs) increase the risk of catheter-related thrombosis; tailoring catheter size to vessel diameter is crucial. The most widely accepted catheter-to-vein ratio is 0.35–0.45 [45,46,47]. Additionally, multi-lumen catheters appear to be associated with a higher risk of catheter-related bloodstream infections than single- or double-lumen VADs due to the complex maintenance that increases the potential for microbial entry. Minimizing the lumen number to single/double lumen catheters can reduce CLABSI incidence [10,48,49]. Infusion of cryopreserved grafts requires large-bore lumens (≥7 Fr for CICC or ≥5 Fr for PICC), controlled manual flow (gravity or syringe; avoid pumps), and immediate saline flushing after each bag; comprehensive protocols are detailed in EBMT/FACT-JACIE standards.

Stem cell transplantation procedures differ in treatment duration, complication risk, infection rates, immunosuppression requirements, and parenteral nutrition needs. In autologous transplantation, particularly for multiple myeloma, peripherally inserted central catheters (PICCs) have demonstrated favorable safety profiles, including low rates of insertion-related complications and catheter-related bloodstream infections (CLABSI) [24,50], as well as improved patient comfort. These findings are primarily supported by retrospective data and institutional experience; direct comparative evidence in HSCT cohorts remains limited.

For allogeneic transplantation with reduced-intensity conditioning (RIC) or non-myeloablative (NMA) regimens, PICCs may represent a feasible option, potentially supplemented by an additional peripheral line for immunosuppressive therapy. This approach could reduce mechanical complications and CLABSI risk while maintaining acceptable catheter-related thrombosis (CRT) rates. However, most supporting data are indirect (general oncology or ICU populations), and recommendations should be interpreted with caution.

In myeloablative conditioning (MAC) settings, centrally inserted central catheters (CICCs) or dual PICCs (inserted bilaterally into two arms) may be considered when high-flow infusions or complex supportive care are anticipated. The role of totally implantable venous access devices (ports) during intensive transplant phases remains uncertain and may be limited to selected low-intensity or outpatient scenarios. Prospective studies reporting outcomes per 1000 catheter-days (CLABSI, CRT, mechanical complications, premature removal, dwell time) are needed to inform evidence-based device selection in HSCT.

### Role of Totally Implantable Venous Access Devices (Ports) in HSCT

Ports (TIVADs) should not be portrayed as universally undesirable in HSCT. Their appropriateness depends on transplant intensity and care setting. During the peak-intensity inpatient phase (particularly allogeneic HSCT or MAC regimens), frequent multi-infusion support (e.g., broad-spectrum antibiotics, transfusion support, possible TPN) and repeated blood sampling favor externally accessible multi-lumen CVCs (tunneled or non-tunneled), which also facilitate immediate removal if CLABSI occurs [10,11,51]. Conversely, outside peak-intensity phases—such as ambulatory care, auto-HSCT pathways with limited concurrent infusions, or post-engraftment follow-up—ports are a reasonable option with favorable complication profiles in oncology cohorts, provided standardized insertion and maintenance bundles are used [6,8,9,10,15]. With respect to HSCT-specific modifiers, allogeneic recipients experience a higher CLABSI burden than autologous programs, supporting preference for multi-lumen, externally accessible CVCs during aplasia [11,52]. Moreover, subclavian access may reduce CLABSI vs. internal jugular in allo-HSCT cohorts without excess mechanical events and can be prioritized when anatomy/operator expertise allows [34]. Parenteral nutrition (PN/TPN) can be delivered via ports with appropriate lumen selection and care (preferably a dedicated access, strict asepsis, and evidence-based flushing/locking), particularly in long-term/home PN settings [5,6,7]. Therefore, the earlier statement that “TPN via ports is problematic” is tempered here to reflect that risks depend on context and protocol adherence. In high-intensity inpatient HSCT—where simultaneous multi-infusion and frequent sampling are needed—tunneled multi-lumen CVCs remain preferable [10,11] (Table 3).

**Table 3:** Suggested device selection by HSCT scenario (with evidence labeling).

Clinical Scenario	Preferred Device	Evidence Type/Key References
Allo-HSCT, MAC, inpatient aplasia (high multi-infusion, frequent sampling)	Tunneled multi-lumen CVC (SCV > IJV when feasible)	HSCT-specific [11,34]
Allo-HSCT, RIC/NMA, ambulatory (lower concurrent infusion demand)	Port or PICC (per center logistics)	Indirect [6,8,9]
Auto-HSCT (outpatient programs), limited concurrent infusions	Port or PICC	Indirect [8,9,49]
Long-term PN after engraftment (ambulatory)	Dedicated port (TIVAD)	Indirect [5,6,7]

Legend: HSCT—hematopoietic stem cell transplantation; MAC—myeloablative conditioning; RIC—reduced-intensity conditioning; NMA—non-myeloablative; SCV—subclavian vein; IJV—internal jugular vein; CVC—central venous catheter; PICC—peripherally inserted central catheter; TIVAD—totally implantable venous access device.

## 8. Complications and Important Clinical Aspects

It is essential to recognize and address all potential complications related to vascular access and maintenance, as these factors directly impact patient outcomes and quality of life. Implementing preventive strategies and promptly managing any challenges are crucial for ensuring successful patient procedures.

### 8.1. Insertion-Related Mechanical Complications

Though preferred in most transplant centers, CICC insertions are bound with significant complications during implantation in 5–19% of cases, such as hemothorax, air embolism, pneumothorax, nerve damage, or tissue hematoma. However, ultrasound guidance has decreased the complication rate to 1–6% [38,53]. TIVAD insertion complications additionally include pocket infection, peri-catheter clotting, and device occlusion [1]. Conversely, PICCs are safe and deprived of the abovementioned complications due to the location of the initial puncture site [24,50]. Preventive strategies are crucial for mitigating complications related to catheter placement. Recent studies and guidelines emphasize the advantages of minimally invasive techniques for CVAD placement, as this approach can eliminate initial complications and improve outcomes and patient comfort. Most studies suggest ultrasound guidance techniques should become the gold standard for all patients [54,55]. The authors of this review recommend that peripheral access should be preferred for stem cell recipients unless contraindicated or clinically insufficient [44].

### 8.2. Catheter-Related Infections

It is essential to note that irrespective of the catheter type utilized, the presence of an indwelling vascular line increases the susceptibility to central line-associated bloodstream infections (CLABSI) [7,56]. The mortality rate related to CLABSI varies between 12% and 40% and depends on patient comorbidities, the specific device employed, and the causative pathogen [57]. Recent data indicate that CLABSIs occur in cancer patients at a rate of 0.5 to 10 cases per 1000 CICC-days [51]. It should be emphasized that the incidence rate of CLABSI is particularly elevated in hematologic patients, reaching 17.3 in aggressive hematological malignancies and 21% during the pre-engraftment phase in patients undergoing HSCT [11,52]. Several retrospective analyses suggest that using peripherally implanted devices could reduce the incidence of CLABSI among this vulnerable population of patients [24]. In a comprehensive comparative analysis conducted by Nakaya et al., it was confirmed that there is a lower incidence of CLABSI in patients with PICCs as compared to those with CICCs. Furthermore, the study showed an interesting microbiological shift, which can be characterized by a decrease in Gram-positive cocci and an increase in Gram-positive bacilli (*p* = 0.001) responsible for CLABSI among patients with PICCs [29].

Similarly, according to the meta-analysis conducted by Schears [28] et al., PICCs are associated with a lower risk of CLABSI compared to CICCs. More precisely, they estimated a significant 48% reduction in CLABSI risk for patients with PICCs compared to CICCs (IRR = 0.52; 95%CI: 0.30–0.92). This might be attributed to a mean rate of 4.09 and 2.12 CLABSIs per 1000 catheter days for CICCs and PICCs, respectively [28]. The authors of this review fully support this approach. Moreover, results of our study revealed that the use of PICCs does not lead to an increase in the incidence rate of CLABSI, regardless of the catheter used [58]. Data regarding PICC-related infections in transplant recipients are predominantly retrospective and scarce, suggesting a CLABSI incidence ranging from 3 to 40% [50,59,60] with a significantly higher rate among allogeneic HSCT, probably as a result of prolonged neutropenia and profound immunodeficiency. It should be emphasized that the incidence of CLABSI, even among central catheters, varies depending on the site of catheter implantation. According to Heidenreich et al., among hematological patients receiving intensive induction or high-dose therapy, CLABSI occurred significantly more frequently when neutropenia lasted longer than 6 days (*p* = 0.024) [33] or the catheter was inserted into the internal jugular vein (IJV) compared to the subclavian vein. This was probably due to the presence of facial hair, neck movements, and worse dressing adhesion [61]. A study conducted by Snarski et al. on behalf of the Infectious Diseases Working Party of The European Society for Blood and Marrow Transplantation (IDWP EBMT) compared the risks of catheter-related infectious complications in two sites of catheter insertion (IJV and SCV) among allogeneic stem cell recipients [34]. The analysis of 232 patients indicated a statistically significant difference favoring the subclavian approach (23% IJV vs. 13% SCV (OR 2.03 (1.01–4.06), *p*  =  0.047)). Following the presented data, in our center, when CICCs are inserted, we favor the subclavian approach to further decrease the infectious complication rate and increase the patients’ comfort.

It is crucial to emphasize that further prospective studies are imperative to provide more definitive recommendations. ESMO, ASCO, and the most recent clinical practice guidelines underscore the significance of implementing clinical bundles, encompassing aspects such as hand hygiene, maximal barrier precautions, chlorhexidine skin antisepsis during catheter insertion, optimal catheter site selection, and assessment of CVAD necessity, to prevent infections. Additionally, it is recommended to use antimicrobial/antiseptic-impregnated and/or heparin-impregnated CVADs to decrease the risk of CLABSI, particularly for short-term CVADs, especially in high-risk groups. However, it is important to note that the prophylactic use of systemic antibiotics is discouraged before insertion [1,2]. Preventive strategies include chlorhexidine-impregnated dressings, strict hub disinfection (‘scrub-the-hub’), and evidence-based lock solutions (heparin, citrate, or ethanol when indicated), with dressing change frequency and hygiene recommendations adapted to neutropenia and mucositis; detailed protocols are provided in EBMT, CDC, and INS guidelines.

### 8.3. Catheter-Related Thrombosis (CRT)

Thrombotic complications can occur regardless of the catheter type used, with reported rates varying from around 5% to an overall rate of 18% [62]. Patients with hematologic disorders, especially those with leukemia, lymphoma, and multiple myeloma, who have indwelling devices are particularly prone to this complication due to frequent immobility, hyperinflammation, and chemotherapy administration. Upper extremity CRT is more common due to the frequent use of CVADs in the subclavian and internal jugular veins. The literature suggests that while TIVADs are generally associated with the lowest risk of catheter-related thrombosis (CRT) [63], evidence regarding PICCs and CICCs appears to favor the latter. A systematic review and meta-analysis conducted by Chopra et al. [64] suggest that PICCs are linked to a higher risk of CRT, although not pulmonary embolism, compared to other tunneled CVADs in cancer patients. It is worth emphasizing that most of the reported data are derived from oncologic patients experiencing prolonged catheterization. The data on CRT in transplantation settings is limited and inconclusive. Stem cell transplant recipients may have altered CRT risk, especially in the case of prolonged and severe thrombocytopenia, which is common in the HSCT setting. Moreover, in the context of stem cell transplantation, catheters are generally removed once therapy is completed.

Results of studies compared by Morano and Fracchiolla have revealed a relatively low incidence of PICC-associated CRT in hematological patients. The exact range varied from 2 to 8%, comparable to conventional central line-associated thrombosis [59,65]. Furthermore, recent studies have revealed a correlation between PICC diameter and thrombotic risk [37,66]. Notably, the data suggest a higher incidence of CRT associated with PICCs than CICCs, particularly for larger-diameter PICCs (>4 Fr). However, the absolute risk of CRT remains low for smaller diameter PICCs, including 4 Fr (<5%). Additionally, single-lumen PICCs appear to be more effective in reducing the risk of CRT than multi-lumen PICCs. Among peripheral devices, current data indicates that polyurethane catheters have an increased rate of CRT but a decreased rate of CLABSI compared to silicone catheters [28,67,68].

The authors of this review recommend implementing a local prophylaxis protocol when using PICCs, along with tailoring devices based on vessel diameter. This strategy appears to effectively decrease the risk of catheter-related thrombosis to that of CICCs, as demonstrated in our comparative analysis [44].

### 8.4. Upper-Extremity CRT with PICCs in HSCT: Evidence, Thresholds, and Risk Mitigation

Evidence synthesis. Contemporary meta-analyses indicate that PICCs are associated with a lower CLABSI risk compared to CICCs but a higher CRT risk, which rises with larger French size and multiple lumens and decreases when single-lumen, small-bore catheters are used [28,64]. HSCT-specific data remain limited but suggest feasibility and acceptable event rates: in allo-HSCT cohorts, CRT incidence was ~9%, with CLABSI ~2.5 per 1000 PICC-days [59]. Our center’s experience confirms that PICC-based strategies do not increase CLABSI when implemented with strict protocols [58], and CRT rates can approach those of CICCs when ultrasound-based selection and catheter-to-vein ratio (CVR) control are applied [44].

Risk modifiers in HSCT. Neutropenia, infection, and immobility amplify CRT and CLABSI risk, while thrombocytopenia complicates anticoagulation decisions without reducing thrombosis risk [62,64]. Device-related factors—French size, lumen number, and insertion site—are major determinants of CRT [64,65].

Practical thresholds and device configuration.

• French size vs. CRT: Symptomatic CRT risk rises steeply with size: 3 Fr~0.9%, 4 Fr~3.3%, 5 Fr~5.5%, 6 Fr~10.7% (oncology data) [64].
• CVR: Maintain ≤ 45% (measured by ultrasound without compression) as recommended by Infusion Therapy Standards [6].
• Lumen number: Single-lumen is preferred; dual-lumen only when clinically essential (e.g., allo-MAC with high infusion demand) [28].

Pharmacologic prophylaxis. Current ASH/ASCO-aligned guidance does not support routine anticoagulation solely for catheter presence; decisions should be individualized based on cumulative VTE risk and bleeding profile [62,64].

Implementation in practice. Our center applies an ultrasound-based selection and maintenance protocol incorporating CVR ≤ 45%, the smallest feasible French size, and minimal lumens. Detailed steps are provided in Supplementary Material S1 [44].

Alignment with 2024 international consensus. The recent oncology consensus emphasizes smallest external diameter, minimal lumens, and bundle adherence, aligning with our approach [4]. However, it does not operationalize CVR thresholds or address HSCT-specific scenarios such as dual PICCs versus CICC in allo-MAC. Therefore, we maintain a nuanced algorithm: PICCs are reasonable in auto-HSCT and allo-RIC/NMA when CVR ≤ 45% and single-lumen designs are feasible, whereas CICC/tunneled lines remain preferred during peak-intensity allo-MAC phases [4,44,59].

Key determinants of CRT risk and evidence tags are summarized in Table 4.

### 8.5. Comparative Summary of Vascular Access Devices in HSCT

To provide a structured overview of vascular access options in hematopoietic stem cell transplantation (HSCT), we present a comparative summary of peripherally inserted central catheters (PICCs), centrally inserted central catheters (CICCs), and tunneled catheters (Table 5). This synthesis integrates key findings from Section 7 and expands upon additional parameters relevant to device selection, including multi-infusion capacity, catheter-to-vein ratio, patient comfort, and feasibility of cryopreserved graft infusion.

Dual PICCs may offer adequate support for multi-infusion therapy in RIC/NMA settings, provided that the catheter-to-vein ratio remains ≤45% and the lumen size is sufficient (≥5 Fr) [6,37]. In MAC settings, CICCs or tunneled catheters are preferred due to higher flow capacity and reduced risk of CRT when multiple upper-extremity devices are avoided [64,65].

Our center employs an ultrasound-based selection and prophylaxis protocol for PICC placement, which has demonstrated CRT rates comparable to CICCs [44]. The cumulative thrombosis risk from multiple upper-extremity devices is mitigated by tailoring catheter size and minimizing total catheter burden [64,66].

**Table 5:** Comparative summary of vascular access devices in HSCT settings.

Parameter	PICC	CICC	Tunneled CVAD	References
CLABSI rate (per 1000 catheter-days)	2.12	4.09	0.1–1.0	[9,28,29]
CRT incidence (%)	2–8% (↑ with >4 Fr, multi-lumen)	5–18%	<5%	[59,64,65]
Mechanical complications	Rare	5–19% (↓ with US guidance)	Moderate	[20,38,53]
Multi-infusion support	Limited by lumen size (4–5 Fr)	Good (multi-lumen, high flow)	Good (multi-lumen)	[69,70]
Cryopreserved graft infusion	Feasible with dual PICCs (≥5 Fr)	Preferred	Feasible	[6,44]
Catheter-to-vein ratio	≤45% recommended	Not applicable	Not applicable	[6,45]
Cumulative thrombosis risk	↑ with multiple PICCs	Lower	Lower	[64,66]
Patient comfort/QoL	High	Lower	Highest	[70,71]
Removal feasibility	Easy	Easy	Surgical, delayed	[16,20]

↓ - decrease; ↑ - increase.

### 8.6. Monitoring of Immunosuppressive Drugs

Therapeutic drug monitoring (TDM) of calcineurin inhibitors during HSCT is critical for efficacy and toxicity control. While venipuncture remains the gold standard, PICC-based sampling is frequently attempted for operational convenience. However, studies indicate potential bias: Shih et al. reported significantly higher cyclosporine concentrations from PICC samples compared to peripheral venipuncture [69], and similar concerns have been raised for tacrolimus [72]. These discrepancies may result from drug adsorption to catheter walls or incomplete clearance during flushing [39].

Standardized sampling protocols are essential to minimize variability. Key elements include:

• Stop infusion time: pause drug infusion for at least 2–5 min before sampling.
• Flush volume: ≥10–20 mL 0.9% NaCl using pulsatile technique.
• Discard volume: at least 2–3 times the catheter dead space (typically 5 mL).
• Dedicated lumen: use a lumen not used for drug infusion whenever possible.
• Documentation: record stop time, flush, and discard volumes in the patient chart.
• If these conditions cannot be met, venipuncture should be prioritized. Operationally, venipuncture ensures accuracy but may increase patient discomfort and staff workload; therefore, a dedicated lumen with strict adherence to protocol can be an acceptable alternative in selected cases. The full recommended protocol and a comparison of venipuncture versus PICC sampling are provided in Supplementary Materials S1 and S2.

### 8.7. Patient Comfort

The ongoing debate regarding the complications and safety of CVAD is deeply entrenched in robust, multidecadal research. Despite this extensive focus on the medical aspects, patient satisfaction and comfort are frequently overlooked in the literature, with only a few studies addressing these concerns. Furthermore, most available data are derived from oncology settings, highlighting a notable gap in research on CVAD-related patient experiences outside of this specific medical context [71,73]. Chen et al. [71] found that the more distal the puncture, the higher the patient comfort, with IJV puncture causing the highest level of discomfort during CVAD implantation. Additionally, a prospective study by Babu et al. [73], which also included hematology patients, highlighted increased comfort from long-term CVAD (CICCs, PICCs, and TIVADs) than repeated cannulation but did not analyze separately the types of VAD included in the study. A study by Fang et al. compared the quality of life (QoL) of patients with all three types of VAD and found that PICCs were associated with higher QoL and greater comfort than CICCs (*p* < 0.01). The superior QoL was noted in TIVADs due to the absence of dressing changes around exit sites, infrequent flushing, lack of visibility of an external line, and minimal risk of catheter malposition [70].

When considering stem cell transplantation, PICCs demonstrate favorable attributes concerning patients’ QoL compared to centrally inserted central catheters (CICCs). PICCs enable increased mobility and decreased limitations in daily activities compared to CICCs. Their placement is less conspicuous, and the dressing can be readily concealed by clothing, which may be preferable for some patients for esthetic reasons. PICCs are generally more accessible and easier to maintain, providing long-term outpatient care. Dressing changes and routine maintenance are typically more straightforward with PICCs, and patients can often be educated to manage certain aspects of their care independently. The authors of this review recommend prioritizing the use of PICCs whenever feasible, as outlined in algorithm No. 1, while considering both patient comfort and procedural safety.

### 8.8. Insertion Site Considerations in HSCT

The selection of a central venous catheter (CVC) insertion site in hematopoietic stem cell transplantation (HSCT) must balance infection risk, mechanical complications, and long-term vascular integrity. Our center favors the subclavian vein (SCV) over the internal jugular vein (IJV) due to lower rates of catheter-related bloodstream infections (CLABSI) and improved dressing adherence, particularly in neutropenic patients. This preference is supported by multicenter prospective data showing significantly reduced infectious complications with SCV access compared to IJV (OR 2.03; 95% CI 1.01–4.06) [34] and by observational studies indicating higher CLABSI rates with IJV due to dressing instability and neck movement [61].

However, SCV access carries a higher risk of pneumothorax, especially in patients with thrombocytopenia or anatomical challenges [38,53]. Additionally, subclavian catheterization may predispose to catheter-associated stenosis, potentially compromising future vascular access in patients requiring long-term therapies or repeat transplantation [20].

Ultrasound-guided techniques, including supraclavicular and low IJV approaches, offer safer alternatives with reduced mechanical complications and improved visualization. These methods may mitigate the infection burden associated with high IJV insertions and reduce the risk of arterial puncture [54,55].

Femoral access devices, including femorally inserted central catheters (FICCs) and femoral ports, are listed among available CVAD types but are not recommended in neutropenic HSCT patients. The femoral site is associated with increased infection risk, poor dressing integrity, and reduced patient mobility. This position is consistent with international guidelines, including ASCO [1], ESMO [2], and the 2024 oncology consensus by Jahanzeb et al. [4], which discourage femoral access in intensively treated cancer patients due to elevated CLABSI risk and compromised care bundles.

## 9. Conclusions

**Figure 1:**
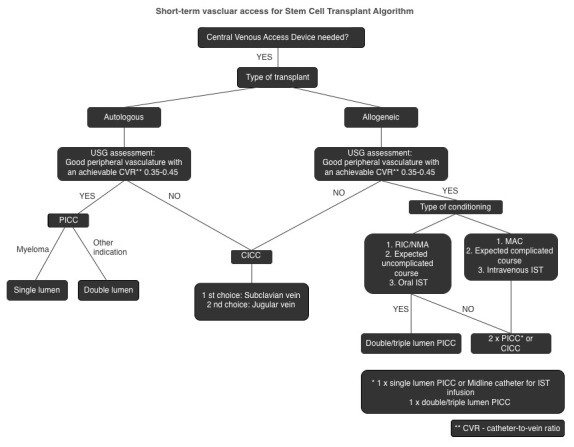
Short-term vascular access for stem recipients. Clinical algorithm. IST—immunosuppressive therapy.

Stem cell transplantation requires versatile vascular access to enable intensive treatment. Furthermore, the comfort of patients should be a paramount consideration within the decision-making framework. In situations where it is clinically viable, the utilization of peripheral access is preferred. This guideline is applicable to both autologous settings and allogeneic transplantation utilizing reduced-intensity conditioning (RIC) or non-myeloablative (NMA) regimens. Conversely, in instances where allogeneic transplantation is conducted using myeloablative conditioning (MAC), the consideration of a tunneled central venous catheter (CICC) or dual peripherally inserted central catheters (PICC) is warranted. CICC is preferred when peripheral vascular access is inadequate, especially with a poor catheter-to-vein ratio, whereas ports (TIVADs) are reasonable outside the peak-intensity phase (e.g., ambulatory auto-HSCT or post-engraftment), provided standardized insertion and maintenance bundles are used; during peak-intensity inpatient phases (particularly allo-HSCT/MAC), externally accessible multi-lumen CVCs remain preferable [6,8,9,10,11,34]. The proposed algorithm is presented in Figure 1.

## Figures and Tables

**Table 1:** Summary of vascular devices used in stem cell transplantation.

Catheter Type	Peripherally Inserted Central Catheters (PICCs)	Tunneled Central Venous Access Devices	Totally Implantable Venous Access Device	Centrally Inserted Central Catheters (CICCs)
Description	Inserted into a peripheral vein and advanced to the central venous system.	Surgically implanted with a portion tunneled subcutaneously before entering a central vein.	A port implanted under the skin connected to a catheter placed in a central vein.	Inserted directly into a central vein (e.g., subclavian or jugular).
Advantages	1. Minimally invasive 2. No significant adverse events upon insertion 3. Suitable for long-term use 4. Facilitates multidrug treatment and TPN administration	1. Lower infection rates compared to non-tunneled catheters 2. Suitable for long-term use 3. Facilitates multidrug treatment and TPN administration	1. Lower maintenance 2. Reduced infection rates 3. Suitable for long-term use	1. Rapid access 2. Suitable for short-term use 3. Facilitates multidrug treatment and TPN administration 4. Accurate immunosuppression levels
Disadvantages	1. Increased risk of thrombosis and occlusion 2. Potential for catheter dislodgement 3. Falsely elevated immunosuppression levels	1. Requires surgical placement and removal 2. Can be uncomfortable for patients 3. Difficult to remove in case of infection	1. Requires surgical insertion and removal 2. Potential for needle dislodgement 3. Difficult to remove in case of infection 4. PN/TPN feasible with a dedicated lumen and standardized maintenance; occlusion risk may increase if flushing/locking is suboptimal [5,6,7]	1. Highest risk of serious mechanical complications upon implantation 2. Higher risk of infection 3. Less comfortable for patients

**Table 2:** Comparative evidence by device type, material, lumen number, and insertion site (HSCT vs. indirect data).

Clinical Determinant	Comparator/Setting	Outcome (Unit)	Effect Size/Absolute Rate	Population/Evidence Tag	Key Sources
PICC vs. CICC	Contemporary practice (meta-analysis)	CLABSI (per 1000 catheter-days/IRR)	IRR 0.52 (95% CI 0.30–0.92)—PICC vs. CICC ↓ infection	Indirect (mixed hospital/oncology)	Schears et al. 2020 [28]
PICC vs. CICC	Adult hematology ward (propensity-adjusted)	CLABSI (per 1000 catheter-days)	PICC 3.29 vs. CICC 5.11; HR 0.48 (95% CI 0.31–0.75)	Indirect (hematology)	Nakaya 2022 [29]
PICC vs. CICC	Contemporary practice (meta-analysis)	Venous thrombosis (CRT/DVT)	RR 2.08 (95% CI 1.47–2.94)—↑ with PICC; attenuation with single-lumen & ≤4–5 Fr	Indirect (mixed)	Schears et al. 2020[28]
Ports (TIVAD)—material	PU vs. SI (forearm ports, large cohort)	Infection/thrombosis; mechanical failure	Complications 1.8 (PU) vs. 0.3 (SI)/1000 cath-days; explantation 10.6% PU vs. 4.6% SI; SI ↑ mechanical failures	Indirect (oncology)	Wildgruber et al. 2016 [30]
Ports (TIVAD)—benchmark	Long-term use	CLABSI (per 1000 catheter-days)	Typowo 0.06–0.30	Indirect (oncology)	Walser 2012 [9] ; Shim 2014 [31]
Ports—number of lumens	Double- vs. single-lumen	Bloodstream infection and dysfunction	HR 2.98 (95% CI 1.12–7.94) for bacteremia (double vs. single); ↑ malfunction/fibrin sheath with double	Indirect (oncology)	Kozlowski et al. 2024 [32]
CICC—insertion site	SCV vs. IJV in hematologic malignancies	CRBSI/CLABSI	CRBSI 1.2 (SCV) vs. 5.7 (IJV)/1000 cath-days; CLABSI 8% vs. 26%; IJV risk ↑ (HR 5.4 for CRBSI)	Indirect (hematology)	Heidenreich et al. 2022 [33]
CICC—insertion site (HSCT)	IJV vs. SCV in allo-HSCT	Infectious complications	OR 2.03 (95% CI 1.01–4.06)—IJV vs. SCV	HSCT-specific	Snarski et al. 2021 [34]
CICC—femoral site	Femoral vs. IJV/SCV (matched analysis)	CRBSI	5.7 vs. 14.2/1000 cath-days; no significant difference short-term	Indirect (oncology)	Hentrich et al. 2023 [35]
Antimicrobial strategies	CHG-alcohol vs. povidone-iodine; antimicrobial/silver-coated CVCs	CLABSI/CRBSI	RR 0.51 (95% CI 0.27–0.97) for CHG vs. PI; antimicrobial coatings reduce CLABSI in networks/RCTs	Indirect (ICU/oncology)	CDC/CLEAN trial [36]; Wang 2018 et al. [25]
Catheter-to-vein ratio	≤45% vs. >45% (upper-arm veins)	CRT	Guideline threshold ≤45% to mitigate CRT; quantitative effect varies by cohort/design	Indirect (standards/ICU)	Gorski et al. 2021 [6]; Zochios et al. 2014 [37]

↓ - decrease; ↑ - increase.

**Table 4:** Determinants of CRT Risk and Evidence Tag.

Determinant	Key Point	Population	Evidence Tag	Sources
PICC vs. CICC—CLABSI	IRR~0.52 (PICC ↓ infection)	Oncology	Indirect	[28]
PICC vs. CICC—CRT	RR~2.0; attenuates with ≤4 Fr, single-lumen	Oncology	Indirect	[28,64]
PICC CRT in HSCT	~9% (allo-HSCT)	HSCT	HSCT-specific	[59]
CVR	≤45% threshold reduces CRT	Practice	Indirect	[6]
Bundle (USG + CVR)	CRT ≈ CICC after implementation	HSCT	HSCT-specific	[44]
2024 Consensus	Smallest size, minimal lumens; no HSCT-specific CVR	Oncology	Indirect	

↓ - decrease.

## Data Availability

Not applicable.

## Supplementary Materials

The following supporting information can be downloaded at: https://www.mdpi.com/article/10.3390/cancers17203325/s1, Material S1: Local Ultrasound-Based PICC Bundle: Step-by-Step SOP; Material S2: Standardized Sampling Protocol for Calcineurin Inhibitor TDM.
