# Supplementary material for: Vascular Access Devices for Stem Cell Transplantation: A Review of Catheter Types—A Crucial Step Towards the Enhancement of Patient Care
Source: Cancers (Basel). 2025 Oct 15;17(20):3325. doi: 10.3390/cancers17203325 (PMC12563463; doi:10.3390/cancers17203325)
Supplement: Supplementary file 1 [file cancers-17-03325-s001.zip › cancers-3882783-supplementary.pdf]

cancers

MDPI

Supplementary Material

Vascular Access Devices for Stem Cell Transplantation: A Review of Catheter Types—A Crucial Step Towards the Enhancement of Patient Care

Sławomir Milczarek, Piotr Kulig, Oliwia Piotrowska, Alina Zuchmanska, Martyna Brzosko and Bogusław Machaliński

Material S1. Local Ultrasound-Based PICC Bundle: Step-by-Step SOP

This section provides a detailed standard operating procedure (SOP) for the local ultrasound-based PICC bundle, including vein mapping, CVR calculation, device selection, tip confirmation, securement, CHG dressings, lock solutions, daily necessity check, and escalation criteria.

Steps:

1. **Pre-insertion ultrasound vein mapping:** Assess basilic > brachial > cephalic veins; measure diameter without compression.
2. **Calculate Catheter-to-Vein Ratio (CVR):**

   CVR = (External catheter diameter ÷ Vein diameter) × 100%; target CVR ≤ 45%.
3. **Device selection:** Choose the smallest feasible French size (typically 3–4 Fr) and minimal number of lumens (prefer single-lumen).
4. **Tip confirmation:** Use ECG-intracavity or radiographic confirmation to ensure correct tip position at the cavo-atrial junction.
5. **Securement:** Apply sutureless securement device and transparent CHG-impregnated dressing.
6. **Lock solutions:** Use appropriate flush and lock protocols (e.g., saline flush after each use; heparin or citrate lock per institutional policy).
7. **Daily necessity check:** Assess ongoing need for the catheter; remove promptly if no longer required.
8. **Escalation criteria:** Immediate removal if severe infection (CLABSI), persistent occlusion, or symptomatic CRT confirmed by imaging.

Material S2. Standardized Sampling Protocol for Calcineurin Inhibitor TDM

Step-by-step protocol:

1. Stop infusion of calcineurin inhibitor for at least 2–5 min.
2. Apply pulsatile flush with 10–20 mL 0.9% NaCl.
3. Discard at least 2–3 times the catheter dead space (minimum 5 mL).
4. Use a dedicated lumen whenever possible; avoid sampling from a lumen used for drug infusion.
5. Collect sample in appropriate tube; label with exact stop time and flush details.
6. Resume infusion only after sample collection is complete.

Comparison: Venipuncture vs. PICC Sampling.

Parameter	Venipuncture (Gold Standard)	PICC Sampling (with protocol)
Accuracy	High	Moderate (protocol-dependent)
Patient comfort	Lower	Higher
Staff workload	Higher	Lower
Risk of bias	Minimal	Present if protocol not followed
